# Web-Based Information Infrastructure Increases the Interrater Reliability of Medical Coders: Quasi-Experimental Study

**DOI:** 10.2196/jmir.9644

**Published:** 2018-10-15

**Authors:** Julian Varghese, Sarah Sandmann, Martin Dugas

**Affiliations:** 1 Institute of Medical Informatics University of Münster Münster Germany; 2 Institute of Medical Informatics European Research Center for Information Systems Münster Germany

**Keywords:** clinical coding, health information interoperability, Unified Medical Language System, eligibility criteria

## Abstract

**Background:**

Medical coding is essential for standardized communication and integration of clinical data. The Unified Medical Language System by the National Library of Medicine is the largest clinical terminology system for medical coders and Natural Language Processing tools. However, the abundance of ambiguous codes leads to low rates of uniform coding among different coders.

**Objective:**

The objective of our study was to measure uniform coding among different medical experts in terms of interrater reliability and analyze the effect on interrater reliability using an expert- and Web-based code suggestion system.

**Methods:**

We conducted a quasi-experimental study in which 6 medical experts coded 602 medical items from structured quality assurance forms or free-text eligibility criteria of 20 different clinical trials. The medical item content was selected on the basis of mortality-leading diseases according to World Health Organization data. The intervention comprised using a semiautomatic code suggestion tool that is linked to a European information infrastructure providing a large medical text corpus of >300,000 medical form items with expert-assigned semantic codes. Krippendorff alpha (K_alpha_) with bootstrap analysis was used for the interrater reliability analysis, and coding times were measured before and after the intervention.

**Results:**

The intervention improved interrater reliability in structured quality assurance form items (from K_alpha_=0.50, 95% CI 0.43-0.57 to K_alpha_=0.62 95% CI 0.55-0.69) and free-text eligibility criteria (from K_alpha_=0.19, 95% CI 0.14-0.24 to K_alpha_=0.43, 95% CI 0.37-0.50) while preserving or slightly reducing the mean coding time per item for all 6 coders. Regardless of the intervention, precoordination and structured items were associated with significantly high interrater reliability, but the proportion of items that were precoordinated significantly increased after intervention (eligibility criteria: OR 4.92, 95% CI 2.78-8.72; quality assurance: OR 1.96, 95% CI 1.19-3.25).

**Conclusions:**

The Web-based code suggestion mechanism improved interrater reliability toward moderate or even substantial intercoder agreement. Precoordination and the use of structured versus free-text data elements are key drivers of higher interrater reliability.

## Introduction

The rise of electronic documentation in health care and research aims to improve patient data exchange not only for proper payment or reimbursement but also for improved data analysis and patient safety; this produces thousands of terabytes of data annually in the United States and Europe [1]. However, ineffective workflows, heterogeneity, and redundancy of data affect the data quality [2-4] and hamper its reuse, comparison, and analysis across different research institutions. Nonstructured and structured data are affected because data elements might be defined or interpreted differently. Semantic coding of data elements enables the identification of semantically matching elements in different data sources and is a key step toward data integration [5,6] and enables the generation of disease-specific core datasets for efficient data capture [7]. Natural Language Processing (NLP) tools use semantic codes for dictionary look-up algorithms or normalize medical terms in clinical free-text notes and use existing semantic thesaurus relations to infer semantic analyses of text segments [8-12].

All of the mentioned examples [5-12] had used the largest clinical metathesaurus available to code medical concepts, the Unified Medical Language System (UMLS) [13], which currently contains >3 million unique concepts; it includes several biomedical vocabularies, for example, clinical reference terminologies as SNOMED Clinical Terms (SNOMED-CT) or Logical Observation Identifiers Names and Codes and medical classifications such as the ICD-10 or other well-known coding systems such as MeSH or MeDRA. Besides the aforementioned advantages of semantic coding of medical content, there is an issue regarding ambiguity in expert-based assignments of such semantic codes—some of the many concepts are synonymous but are given different concept identifiers; for example, the UMLS concept “antidementia drug” (Code: C1276997) and the concept “antidementia agents” (Code: C1531592) are synonymous from a clinical point of view but are represented by 2 different codes. In addition, there is a low semantic similarity or relatedness between those concepts based on the ontological structure of UMLS source vocabularies [14].

In practice, synonymy and abundance of very similar but different concepts in some clinical subdomains lead to inconsistent coding among different coding experts, which weakens the advantage of semantic coding to improve data integration. NLP or information extraction tools that use UMLS as core terminology are affected as well because their programmed assumption “Different UMLS codes represent semantically different concepts” is flawed. Moreover, the evaluation of different NLP tools is challenging, that is, if 2 NLP tools A and B suggested 2 different UMLS codes for a text segment, it may not be clear whether the output of A or B is more valid.

Low rates of human expert-based interrater reliability have been evaluated for the clinical terminology SNOMED-CT [15,16]. Rothschild et al [17] reported that interrater reliability is moderate at best with UMLS in unstandardized problem lists and suggested that coder training and standardization might improve interrater reliability. Our work provides a significant novelty by performing a systematic interrater reliability analysis of UMLS on several mortality-leading disease domains and conduction of an interventional pre-post study. The intervention included a coder training for using a Web-based semiautomatic code suggestion tool that utilizes a large metadata repository as an expert coding knowledgebase to improve uniform coding among different raters. Following key questions formed the rationale of this study:

1. What is the effect of the intervention on interrater reliability?
2. How does interrater reliability differ when coding structured data elements versus free-text?
3. How does interrater reliability relate to precoordinated versus postcoordinated concepts?

Question 1 seeks to improve interrater reliability by systemizing the way a coder uses a large metadata registry to reuse common precoded medical concepts. This way, a coder would be suggested a preferred UMLS code based on the coding frequency of other expert coders who have already coded the same or similar portion of text.

As for question 2, it is well known that structured data elements are more suitable for data exchange across different information systems than free-text in clinical reports or eligibility criteria of clinical trials [18]. However, free text is still existing and necessary in medical documentation. Both structured data elements and free-text elements could be semantically annotated to foster semantic interoperability. Therefore, our study measured interrater reliability in structured routine documentation forms versus free-text eligibility criteria to examine interrater reliability differences depending on both types of documentation.

Regarding question 3, to code medical concepts of medical data elements or free text, one distinguishes 2 basic semantic coding methods, called precoordination and postcoordination [19]. A medical concept is precoordinated if its semantics are represented by one semantic code; for example, the term “Patient has diabetes mellitus type 2” contains the medical concept “diabetes mellitus type 2” and can be coded by a single UMLS concept code “C0011860-Diabetes Mellitus, Non-Insulin- Dependent.” A medical concept is postcoordinated if it is coded by multiple codes to express more complex semantics; for example, the term “Patient has an allergy to Amoxicillin” contains the medical concept “Allergy to Amoxicillin” and could be coded by the following 2 interrelated UMLS codes: “C0020517-Hypersensitivity” and “C0002645- Amoxicillin.” Another type of coding that can be considered as a special type of postcoordination is the coding of multiple separated medical concepts in one medical term; for example, “Patient has an allergy to Amoxicillin or has diabetes mellitus type 2” contains 2 preceding medical concepts and could represent a free-text inclusion criterion in clinical trials.

The assessment of semantic coding correctness among different coders was not the scope of this analysis and would be pointless because coders recruited for the studies were considered as medical terminology experts. Therefore, the key challenge for accurate UMLS coding is to achieve high interrater reliability among different coders rather than finding a semantically correct coding.

To address these 3 key questions, a quasi-experimental study was conducted with medical experts as study subjects to report on the effects on the coding behavior with and without a Web-based coding suggestion tool.

## Methods

### Information Infrastructure and Recruitment

The Medical Data Models (MDM) portal is a Web-based large, open-access, metadata registry and European information infrastructure [20] funded by the German Research Federation. More than 15,000 medical forms with >300,000 form items are available; all of them are UMLS coded by medical experts. Each expert undergoes training on how to use an expert-based code suggestion mechanism [5] within the MDM portal; by this, each new coder can choose from previously coded concepts if similar item text patterns exist. To increase the throughput of coded items for the MDM project, 6 final-year medical students from the medical faculty in Münster, Germany, were recruited. None of the students had any experience in UMLS coding. Interrater reliability was assessed before and after the training to address the aforementioned study key questions.

### Study Setting and Material

**Figure 1:**
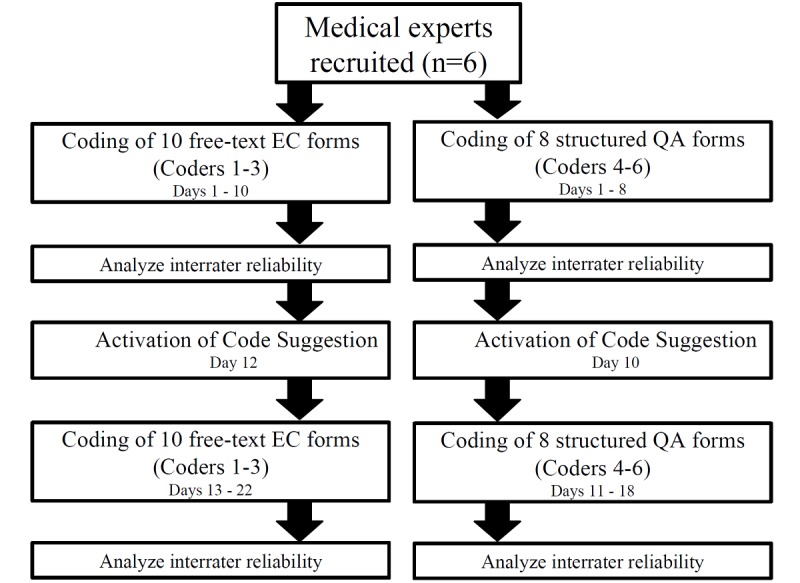
The study workflow. EC: eligibility criteria; QA: quality assurance.

The study is a pre-post analysis that was conducted from February 15, 2017 to March 12, 2017, at the Institute of Medical Informatics, University of Münster (Münster, Germany). Figure 1 illustrates the flowchart of the study design; each coder coded a single form per day and days are regarded as consecutive working days.

We randomly selected 10 eligibility criteria forms of different clinical trials (conducted between 2000 and 2016) from ClinicalTrials.gov. Based on the manual review, each form was only selected if its study was related to a medical condition that was a leading cause of death based on the World Health Organization 2015 Global Health estimate data [21]. If a form did not adhere to this, it was discarded, and the next form was considered until a set of 10 forms was selected. Then, 8 quality assurance forms were analogously collected for the preinterventional phase to provide a dataset of structured documentation forms with a similar number of items. The quality assurance forms originated from the Institute for Applied Quality Improvement and Research in Health Care [22] in Germany and Austria and are implemented by law in all hospitals of Germany that provide therapeutic procedures, which are under governmental quality assurance [23,24]. These forms contain a series of structured routine documentation items, including quality indicators before, during, and after health care procedures.

**Table 1:** The disease category coverage in eligibility criteria forms and quality assurance forms.

Disease category	Documentation models to code (number form models)			
	Eligibility criteria pre-intervention	Eligibility criteria post-intervention	Quality assurance pre-intervention	Quality assurance post-intervention
Cardiovascular (including myocardial infarction and stroke)	4	4	2	2
Respirational diseases	3	3	1	1
Diabetes mellitus and pancreatic diseases	1	1	1	1
Renal diseases	0	0	1	1
Liver diseases	0	0	1	1
Breast cancer	0	0	1	1
HIV/AIDS	1	1	0	0
Traumatic or orthopedic diseases	1	1	1	1

**Figure 2:**
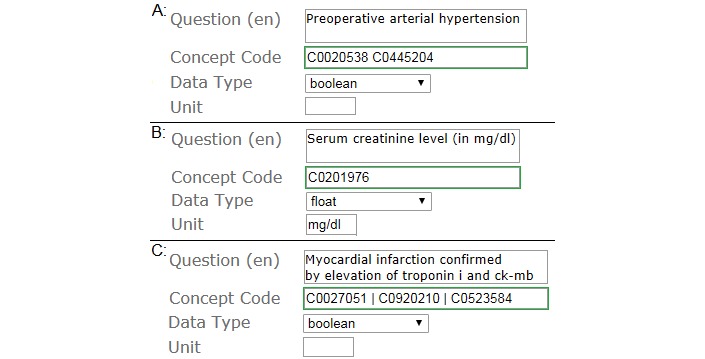
Item coding view in ODMEdit.

For every form in the preinterventional phase, a different disease-matching form was collected for the postinterventional phase. Therefore, 36 forms (2×10 eligibility criteria forms+2×8 quality assurance forms) were included in total for this study. Table 1 summarizes the medical condition categories.

All selected forms were transformed to the Operational Data Model (ODM), an international standard by the Clinical Data Interchange Standards Consortium [25] for the representation and exchange of clinical trial data and metadata. In addition, semantic coding was added using ODMEdit [5]—a Web-based app, which is accessible from the MDM portal and features the semiautomatic code suggestion based on coded items in the MDM database. Multimedia Appendix 1 provides the full set of all study forms (eligibility criteria + quality assurance forms) as Clinical Data Interchange Standards Consortium ODM files. Figure 2 shows screenshots of 3 exemplary items in the item coding view that every coder used. All 3 items covered the 3 aforementioned types of concept coding (precoordination, postcoordination, and multiple concept coding). Figure 2 shows the quality assurance item that needed postcoordination (2 codes to represent one complex concept: preoperative arterial hypertension) and the quality assurance item that was coded with one precoordinated concept with one Unified Medical Language System code: C0201976—Serum creatinine level. Measurement units, data type, or permissible values were not the scope of semantic coding. It also shows the eligibility criteria form item expressing an eligibility criterion of a clinical trial that was coded with 3 codes to express 3 different medical concepts (myocardial infarction as a diagnosis, troponin I, and ck-mb as laboratory markers).

All 6 recruits agreed to participate and were then randomly assigned to code eligibility criteria forms of clinical trials or quality assurance forms. In the preinterventional phase, all coders received a basic 15-minute introduction to use the standard UMLS metathesaurus browser (version 2015AB) to understand the concepts of pre- and postcoordination and use ODMEdit for semantic annotation of form items. In addition, they were instructed to UMLS code-relevant medical concepts of their given form items. Relevant medical concepts were defined to be concepts that the coder deemed significant to capture the semantics of the given form item. Of note, UMLS coding was restricted to the concept domain of possible medical data elements; it does not concern the coding of the value domain. For instance, in the term “Creatinine value of >7 mg/dL,” “Creatinine” is the medical concept of the concept domain; “>7 mg/dL” represents the corresponding value and measurement unit and, therefore, is not considered for the coding procedure. The use of the integrated code suggestion function—which is the key part of the intervention—was prohibited in the preinterventional phase. All recruits performed UMLS coding at their homes using a broadband internet connection to access the MDM portal.

### Study Intervention

The intervention of this study consisted of a 60-minute coder training to teach the use of ODMEdit’s code suggestion function and basic coding principles [18]. Each training session consisted of one participant and the same supervisor with extensive experience in UMLS coding in structured data elements [7] and free-text eligibility criteria [18]. The details of semiautomatic ODMEdit’s code suggestion tool are described in our previous work [5]. Multimedia Appendix 1 provides the full teaching material that was provided to the coders.

Each coder was free to follow the coding suggestion (or not). None of the study forms were part of the training, and all participants were prohibited from sharing or discussing their coded forms during the study period. None of the 36 study forms existed in the MDM database before the start of the study. If a coder did not find a suitable code through the suggestion function, the code search could be extended with the standard UMLS metathesaurus browser. Notably, no time restriction was applied in the pre- and postinterventional phase. If coders ended up with no suitable code for a form item, it was up to them to skip the item and move on to the next one.

### Interrater Reliability Measure and Coding Time

UMLS codes represent nominal data. The interrater reliability statistics as the simple calculation of percentages of the observed agreement are associated with biases and should be corrected for the agreement expected by chance [26]. Further measures as Cohen kappa [27] are restricted to the use of 2 raters or other limitations [28-31], whereas Krippendorff alpha (K_alpha_) [32] with bootstrap CIs is reported as recommended especially in cases of missing data—compared with Fleiss K—with >2 raters and with a large amount of different rating categories [31]. Landis and Koch [33] proposed the following interpretation regarding K_alpha_ value ranges: <0, poor agreement; 0.00-0.20, slight agreement; 0.21-0.40, fair agreement; 0.41-0.60, moderate agreement; 0.61-0.80, substantial agreement; and 0.81-1.00, almost perfect agreement.

K_alpha_ was calculated among all 3 raters in all 4 subgroups as follows: preinterventional eligibility criteria forms; postinterventional eligibility criteria forms; preinterventional quality assurance forms; and postinterventional quality assurance forms. The coding of a form item between 2 coders is only considered to be matching if the set of UMLS codes were the same. The calculation was performed with the R-package by Zapf et al [31]; this package also includes bootstrap analysis to determine CIs using 10,000 bootstraps.

Each coder measured his or her coding time via stopwatch by starting the time before the first item and stopping after the last item of a form. Thus, data on the mean coding time per item for each form are available but without item-based time variances. The median coding time per item was then determined for each user as the median mean coding time per item of all forms before and after the intervention.

Of note, this study did not intend to analyze the intervention as a cause of coding time differences. For this purpose, a different study design would have been appropriate. Instead, this study was designed to primarily analyze the effects on interrater reliability. However, time measurements were still taken to audit any adverse coding time expenditures associated with the intervention. As each coder could potentially remember same or similar items from previous forms, a learning effect could bias the time measurements for consecutive forms. To account for this issue, time measurements will be presented for each coder coupled with a learning graph to illustrate the number of new medical concepts for each coding day of the study.

## Results

### Effect of the Intervention on the Interrater Reliability and Coding Time

K_alpha_ increased for both documentation types (structured quality assurance and free-text eligibility criteria). A significant difference with respect to 95% CIs existed within the eligibility criteria study group (0.43, 95% CI 0.37-0.50 vs 0.19, 95% CI 0.14-0.24). The median word counts per item were comparable for pre- and postinterventional form item sets based on the interquartile ranges (see Table 2 for details).

**Figure 3:**
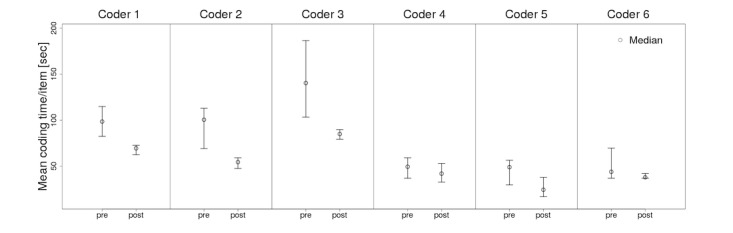
The mean coding times per item for each rater before and after the intervention.

**Figure 4:**
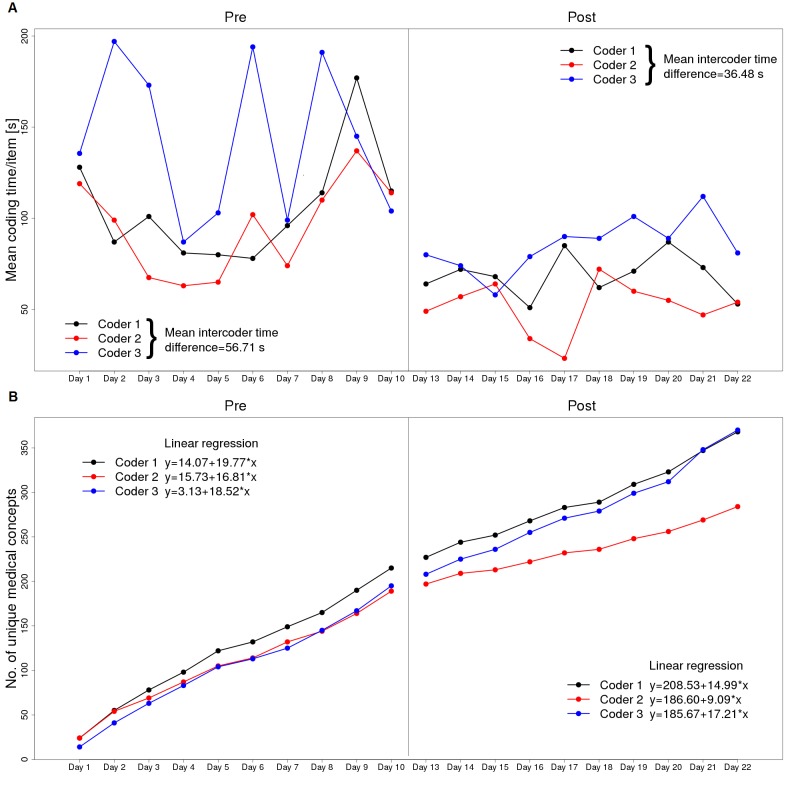
A: The mean coding times for eligibility criteria forms before and after the intervention. B: The number of unique medical concepts each coder has coded on each day.

**Figure 5:**
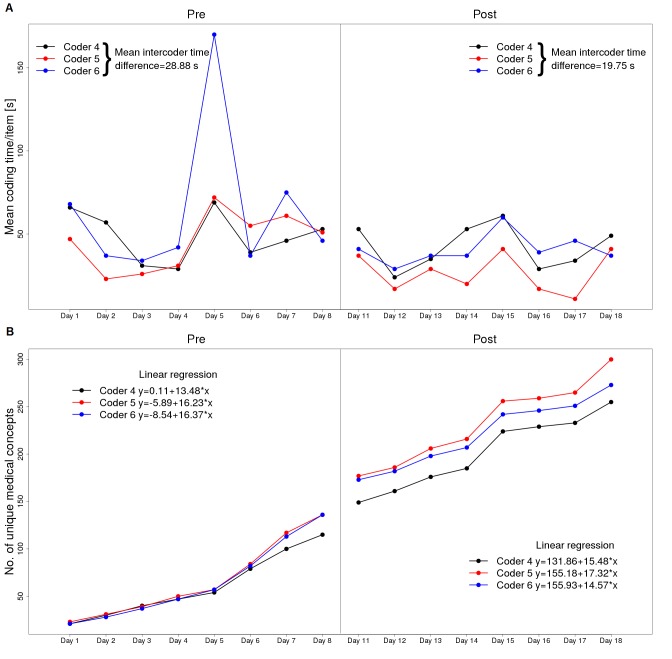
A: The mean coding time per item. B: Daily new unique concepts to code for structured quality assurance forms, analogous to Figure 4.

Based on time measurements for each coder, median coding time per item was decreased in all 6 raters but with overlapping interquartile ranges in the quality assurance subgroup, as shown in Figure 3; it includes the median with interquartile ranges is calculated on the basis of mean coding time per items of all forms before and after the intervention; coders 1-3, free-text eligibility criteria study group and coders 4-6, structured quality assurance forms. Figures 4 and 5 illustrate the coding time during the course of the full study coupled with the aforementioned graph of newly coded concepts on each day for eligibility criteria and quality assurance forms, respectively. On each day, identical form items were coded among different coders. The mean intercoder time difference averages absolute time differences between all 3 coders on each day, as shown in Figure 4; it also includes the interception and slope of each coder-related learning graph is calculated on the basis of linear regression. Within the preinterventional eligibility criteria forms, the median number of new medical concepts among all raters was 20 per day (interquartile range, IQR 16.25-24). After the intervention, it reduced to 13 (IQR 9.25-17).

**Table 2:** The effect of the intervention on the interrater reliability.

Coded models		Preintervention			Postintervention		
		Number of items	MWC^a^ (IQR^b^)	K_alpha_^c^ (95% CI)	Number of items	MWC (IQR)	K_alpha_ (95% CI)
**Free-text eligibility criteria forms**		142	10 (6-14)	0.19 (0.14-0.24)	150	9 (4.5-13.5)	0.43 (0.37-0.50)
	Precoordinated item set	20	3 (2.00-5.25)	0.64 (0.46-0.79)	67	5 (4.00-9.00)	0.78 (0.70-0.86)
	Postcoordinated item set	122	10 (6-14)	0.12 (0.08-0.16)	83	11 (8-17.5)	0.16 (0.11-0.21)
**Structured quality assurance forms**		159	3 (2-4)	0.50 (0.43-0.57)	151^d^	3 (2-4)	0.62 (0.55-0.69)
	Precoordinated item set	102	3 (2.00-4.00)	0.72 (0.64-0.80)	116	3 (2.00-4.00)	0.76 (0.69-0.82)
	Postcoordinated item set	57	5 (3.00-6.00)	0.12 (0.07-0.15)	33	4 (2.00-6.00)	0.15 (0.07-0.23)

^a^MWC: median word count per item.

^b^IQR: interquartile range.

^c^K_alpha_: Krippendorff alpha based on 10,000 bootstrapes (95% CI).

^d^Two items could not be coded by any of the coders.

Regarding quality assurance forms, the medians remained the same or slightly increased after the intervention (13, IQR 8.75-23 vs 13.5, IQR 8.25-34.25); this is in accordance to linear regression applied on each of the learning graphs in Figures 4 and 5 (see slope changes before and after intervention). Regarding intercoder time comparisons, the mean intercoder time differences decreased after the intervention in both documentation types (see Figures 4 and 5).

A form item is counted as precoordinated if each coder picked one single (not necessarily the same) Unified Medical Language System code.

### Free-Text Eligibility Criteria Versus Structured Quality Assurance Items

Before and after the intervention, structured quality assurance items were associated with significantly higher interrater reliability than free-text eligibility criteria items. However, after the intervention, the difference narrowed down because of a stronger interrater reliability increase in the free-text eligibility criteria items set (before intervention: K_alpha_=0.17 in eligibility criteria and K_alpha_=0.50 in quality assurance; after intervention: K_alpha_=0.42 in eligibility criteria and K_alpha_=0.62 in quality assurance); see Table 2 for details.

### Precoordinated Versus Postcoordinated Concepts

**Figure 6:**
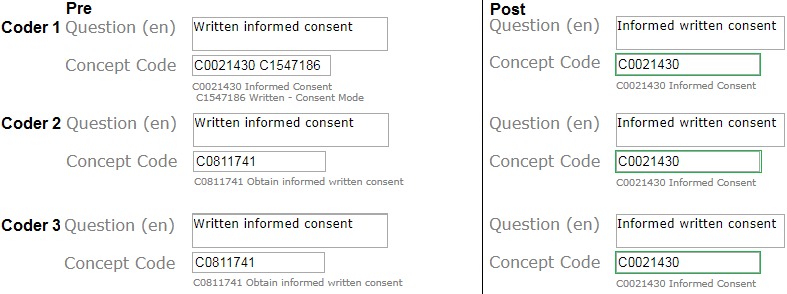
An example of coding harmonization after intervention.

Interrater reliability was significantly higher in precoordinated items versus postcoordinated items before and after intervention regardless of the documentation type. Precoordinated items had an interrater reliability with K_alpha_ ranging from 0.64 to 0.78, and postcoordinated items had an interrater reliability with K_alpha_ ranging from 0.12 to 0.16 (see Table 2 for further details). The coder’s decision to pre- or postcoordinate significantly changed after the intervention. The proportion of items that were precoordinated significantly increased both in the eligibility criteria and quality assurance item set (eligibility criteria: OR 4.92, 95% CI 2.78-8.72; quality assurance: OR 1.96, 95% CI 1.19-3.25). Figure 6 provides an example of 2 similar eligibility criteria form items that were coded by different coders to illustrate coding harmonization after intervention. Before intervention, the study inclusion criterion “Written informed consent” was coded the same among coders 2 and 3. Coder 1 used postcoordination with different codes. After intervention, all 3 coders coded the semantically identical inclusion criterion “Informed written consent” using a simplified precoordinated common medical concept, Informed Consent with Unified Medical Language System code C0021430, which was suggested by the code suggestion mechanism and covers sufficiently enough the relevant meaning of inclusion criterion, as shown in Figure 6.

## Discussion

### Interrater Reliability

Before the intervention, interrater reliability was low or moderate, as reported in related work for other terminologies, such as the emerging clinical reference terminology SNOMED-CT [15,16]. To the best of our knowledge, this is the first study to evaluate the increased interrater reliability through activation of the expert-based code suggestion, which is linked to a large repository of already annotated medical items. Other large terminologies that also suffer from low interrater reliability because of the high abundance of similar or duplicate concepts could benefit from our approach as well.

In this study, the relative increase was more pronounced in free-text eligibility criteria coding. One key observation could explain this phenomenon in the subgroup analysis; owing to the high number of words in an eligibility criterion, coders frequently had chosen postcoordination with rather a word-by-word coding than to search for a common single medical concept to capture the relevant semantics of the whole eligibility criterion. With postcoordination, the probability that another coder chooses the same sequence of codes decreases with each additional postcoordinated code. After the intervention, the coders chose precoordination markedly more often over postcoordination. Although the preference of precoordination is explicitly mentioned as a general rule of thumb as part of the coder training (based on the established coding principles [18]), the code reuse function identifies semantically similar medical text items from a large semantically annotated text corpus and suggests simplified precoordinated coding even for complex free-text items (see example in Figure 6). Because the code suggestion function and the training of coding principles form one coherent unit of the intervention, we did not intend to analyze both parts separately, for example, by further study arms. Therefore, this intervention has to be taken as a whole with respect to reported effects.

According to the K_alpha_ value interpretation by Landis and Koch [33], interrater reliability improved to “moderate agreement” (at least fair agreement regarding 95% CI) and “substantial agreement” (at least moderate agreement regarding 95% CI) in the free-text eligibility criteria set and structured quality assurance set, respectively. The perfect agreement would be required for automatic comparisons based on sole UMLS codes. Therefore, expert-based code review in cases of disagreements might be necessary to rule out false-positive disagreements.

### Coding Time

Time measurements indicate slight reductions in coding time in the eligibility criteria subset and a similar coding time in the quality assurance subset. There was a substantial decrease in new concepts in the postinterventional phase for the eligibility criteria set compared with that in the quality assurance set in which the median number of new concepts barely changed; this difference was expectable because the disease-matched eligibility criteria forms were chosen from different clinical trials but they do contain similar inclusion and exclusion criteria. Unlike the eligibility criteria forms, the quality assurance forms stemmed from one source responsible for nationwide quality assurance documentation and, therefore, repetitive data elements among different forms were less common than that in eligibility criteria forms.

### Translation Into Natural Language Processing-Based Use Cases

NLP tools that rely on expert-annotated medical text for training can take advantage of this large data repository. As the largest repository of medical data items with semantic codes, it currently consists of >300,000 English medical form items, which are semantically annotated by medical experts with a broad coverage on diverse disease entities [20]. Thus, the meaning of diverse medical text segments, including synonyms and complex clinically relevant concept relations, is machine readable. The study has shown that the use of this large data repository and coding principles improved uniform (=high interrater reliability) coding among different human coding experts. Because NLP pipelines and machine-learning approaches, in general, use expert-annotated text corpora with information coded by different experts, higher interrater reliability would increase the signal-to-noise ratio and, thus, improve semantic classification accuracy in natural free-text. In turn, NLP tools could be more effective in the identification of clinically relevant concepts hidden in clinical notes and corresponding biomedical literature and could be linked to computerized decision support systems for the implementation of evidence-based management strategies at the point of care [34].

### Limitations

This study is the first to analyze the effect among different medical coders before and after a training intervention. To the best of our knowledge, a larger set of medical expert coders were never recruited for systematic UMLS intercoder analyses. A larger set of coders with an even larger number of form items to code would have been beneficial to limit the range of dispersion for the reported CIs. However, the strength of the sample size lies in the unprecedented high number of 602 different form items (EC forms pre+post, 292 items; quality assurance forms pre+post, 310 items) covering a broad area of mortality-leading diseases and showing statistical significance in the free-text coding task.

This study had a quasi-experimental design. A randomized controlled design would provide the gold standard to elaborate on the cause and effects of the reported intervention. However, in this study, randomization would have decreased the number of participants assigned to the intervention arm to only 3 participants, whereas the more simplistic pre-post design had the advantage of evaluating all 6 coders with and without intervention and intracoder-related effects (interrater reliability, decision to precoordinate, and coding time) during the full course of the study.

The form content was selected on the basis of the availability and the leading causes of World Health Organization mortality data and, therefore, addresses a broad range of medical concepts in disease entities, which are of high research-interest or under quality assurance. However, the reported effects on interrater reliability might not be generalizable to other clinical fields.

A conceivable success factor of the semiautomatic code suggestion is the underlying annotated text corpus in the MDM portal having oncology as the major disease entity as form content and not mortality-leading diseases such as cardiovascular diseases [20]. Currently, a continuous development of the annotated text corpus is ongoing [35] in many different medical fields and could, therefore, yield a further increase in the interrater reliability in the future.

### Conclusions

Coder training and Web-based semiautomatic code suggestion improved interrater reliability in coding medical concepts of diverse mortality-leading disease areas while preserving or even slightly decreasing coding time. Higher interrater reliability represents higher coding uniformity among different medical coders. Consequently, this would lead to a higher signal-to-noise ratio in use cases, which utilize text corpora annotated by multiple coders for semantic analyses.

This study indicates that precoordination in preference to postcoordination and the use of structured data elements in preference to free-text data elements are key drivers for higher interrater reliability. Further development of not only the code suggestion mechanism and use-case specific coder training but also harmonization of codes in the provided medical terminology system are necessary to achieve substantial or almost perfect agreement consistently.

## Appendix

Multimedia Appendix 1 Coded forms and teaching material.

## Abbreviations

IQR: interquartile range
UMLS: Unified Medical Language System
K_alpha_: Krippendorff alpha
MDM: Medical Data Models
NLP: Natural Language Processing
ODM: operational data model
