# Supplementary material for: Web-Based Information Infrastructure Increases the Interrater Reliability of Medical Coders: Quasi-Experimental Study
Source: J Med Internet Res. 2018 Oct 15;20(10):e274. doi: 10.2196/jmir.9644 (PMC6231825; doi:10.2196/jmir.9644)
Supplement: Multimedia Appendix 1 [file jmir_v20i10e274_app1.zip › ExtendedCodingPrinciples.docx]

**Coding Principles Extension**

The following guidance table extends the UMLS coding principles published by Varghese et al. in 2015 [1] and lists frequent coding issues that were experienced since then and provides corresponding guidance to solutions.

| Coding Principle Extension ID | Coding Issue | Guidance Description |
| --- | --- | --- |
| CPE.1 | General question of relevance: Should the term be coded at all? | -Yes, if it is a technical term or commonly used concept that represents clinically relevant decision parameters in health care or in the current coding context. |
| CPE.2 | Post-coordination syntax | Post-coordination is expressed by combining several codes to represent a complex medical concept (e.g. „Allergy to amoxicillin”).  General type of post-coordination: The first code represents the main concept, while all further attached codes are used as additional qualifiers to specify coding. A special type of post-coordination is the coding of multiple different medical concepts in one term ( e.g. „pregnant or breastfeeding”, could also be understood as multiple pre-coordination per term).  An exemplary syntactical expression using Operational Data Model by CDISC for both types:  1)  <ItemDef OID="I.1" Name="Allergy to amoxycillin" DataType = "boolean">  <Question>  <TranslatedText xml:lang="en">Allergy to amoxicillin ? </TranslatedText>  </Question>  <Alias Context="UMLS CUI [1,1]" Name="C0020517"/>  <Alias Context="UMLS CUI [1,2]" Name="C0002645"/>  </ItemDef>  2)  <ItemDef OID="I.2" Name="pregnant or breastfeading" DataType="boolean">  <Question>  <TranslatedText xml:lang="en">Pregnant or breastfeading ?</TranslatedText>  </Question>  <Alias Context="UMLS CUI [1]" Name="C0032961"/>  <Alias Context="UMLS CUI [2]" Name="C0006147"/>  </ItemDef> |
| CPE.3 | How to simplify terms to be coded | Simplify coding by identifying the main concept and search that concept within the MDM portal and its code suggestion tool [2, 3]. If frequency is low: choose an equivalent concept name. Bare in mind: Common terms are coded in the Mdm-knowledge base. |
| CPE.4 | In General, terms should be coded with high specificity. However, how to deal with ambivalence between concept **a** and more=too specific concept **b**, which one to choose? | 1): Preference towards frequency of existing concept names based on MDM [2, 3] portal (code suggestion function)  2): In case of even heterogeneity/entropy in code suggestion: Take the more general concept. In case of is-relations: Post-coordinate the specific qualifier to improve data integration and comparability with similar concepts. |
| CPE.5 | Pre- vs Post-coordination | Prefer a pre-coordinated code if the term (possibly consisting of several medical terms) represents a clinically relevant or frequent concept as a whole. Use post-coordination for minor additional concepts (localization and qualifiers such as severe, mild or less) OR clinically relevant binary/ternary relations: E.g. Drug allergy, Drug interactions, Therapy Contraindications |
| CPE.6 | Should the term “patient” be coded? | All assigned codes are assumed to bepatient-centered: E.g. "patient height" will only be coded by the concept "Body height"(C0005890). If there is a concept related to another person, a further code is post-coordinated for that person: E.g. "diagnosis of mother": use code for "mother" and "diagnosis" |
| CPE.7 | Concept domain coding | The scope of these coding principles refer to the concept domain not on temporal information or measurement units (esp. lab values!) or other value domains. |
| CPE.8 | Logical operators | Ignore logical operators that link two main concepts (e.g. “pregnant or lactating”. Negations of qualifier concepts should coded. (E.g. “Reason for not having obtained X” -> apply post-coordination: Code_Reason + Code_Not_obtained) |
| CPE.9 | Vague organ functions or medical conditions? | Prefer to code objective measurements such as lab values (if given) instead of vague organ function terms (e.g. “renal insufficiency with creatinine > 1.1 mg/dl” -> code “serum creatinine”) |
| CPE.10 | Classification items | Prefer to code the classification names and not classication items themselves, e.g. "Patient is in NYHA 2" -> code “NYHA classification” and not “NYHA 2” (Principle of coding the concept and not the value domain) |
| CPE.11 | Concepts accompanied with basis medical processes terms | Avoid coding medical process steps for terms like "having administered", "treated", "diagnosed"  -> For terms like "Patient has Diagnosis X" -> code the disease X! |

**References**

1. Varghese J, Dugas M et al. Frequency analysis of medical concepts in clinical trials and their coverage in MeSH and SNOMED-CT. Methods Inf Med 2015; 54(1):83–92.

2. Dugas M, Meidt A, Neuhaus P, Storck M et al. ODMedit: uniform semantic annotation for data integration in medicine based on a public metadata repository. BMC Med Res Methodol 2016; 16:65.

3. Dugas M, Neuhaus P, Meidt A, Doods J et al. Portal of medical data models: information infrastructure for medical research and healthcare. Database (Oxford) 2016; 2016.
