# Supplementary figures and images for: Web-Based Information Infrastructure Increases the Interrater Reliability of Medical Coders: Quasi-Experimental Study
Source: J Med Internet Res. 2018 Oct 15;20(10):e274. doi: 10.2196/jmir.9644 (PMC6231825; doi:10.2196/jmir.9644)

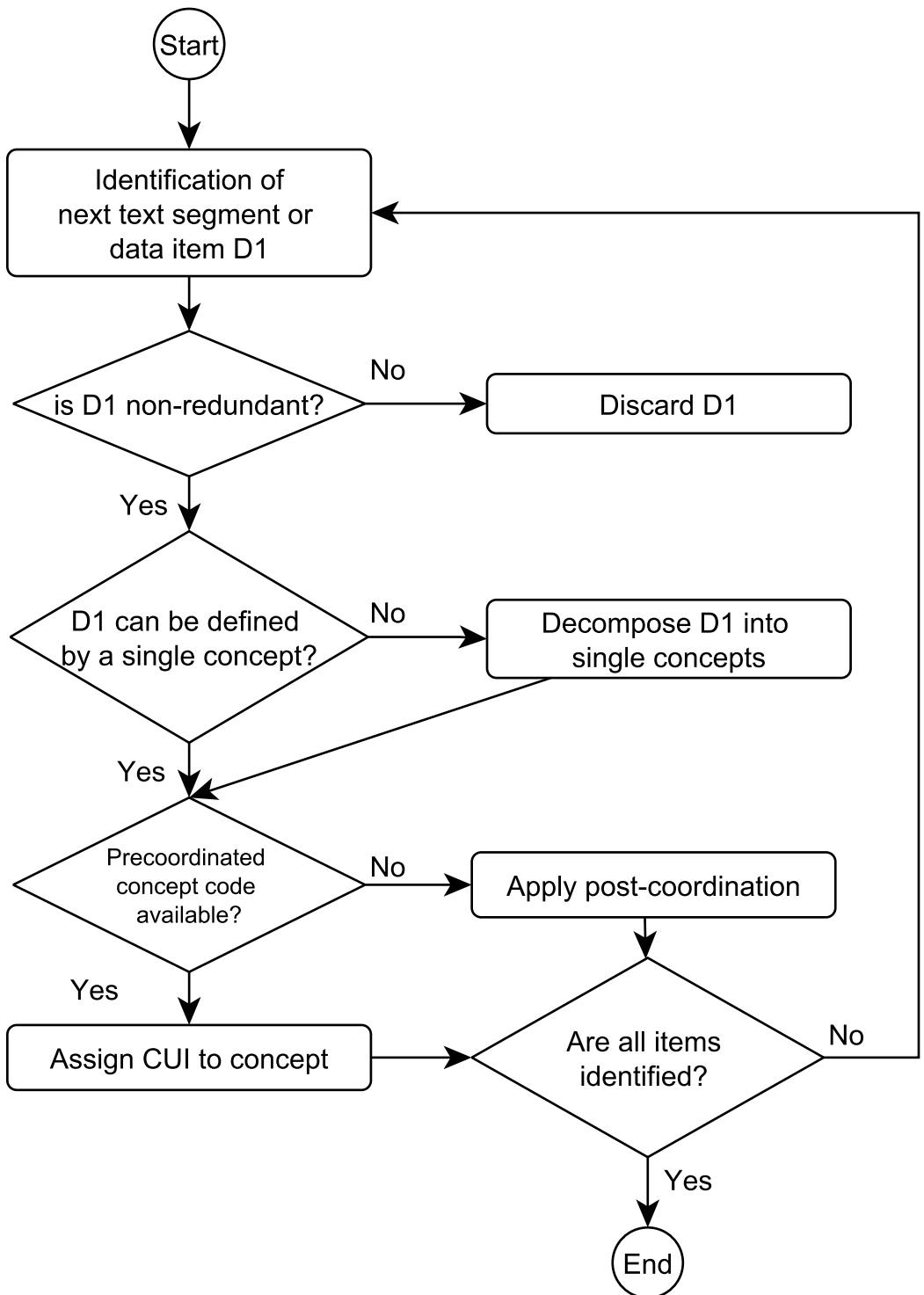

Supplement: Multimedia Appendix 1 [file jmir_v20i10e274_app1.zip › FlowChart-BasicCodingPrincples.pdf]
